# Suicidal behavior in combat veterans with mood disorders

**DOI:** 10.1192/j.eurpsy.2024.101

**Published:** 2024-08-27

**Authors:** L. Sher

**Affiliations:** ^1^James J. Peters VA Medical Center; ^2^Icahn School of Medicine at Mount Sinai; ^3^Columbia University Vagelos College of Physicians and Surgeons, New York, United States

## Abstract

**Abstract:**

**Introduction.** Military conflicts are ubiquitous. There are many combat veterans around the world. The combat environment is characterized by violence, physical strains, separation from loved ones, and other hardships. Mood disorders and suicidality in combat veterans are a large and important issue.

**Objectives:**

To discuss the pathophysiology and prevention of suicidal behavior in combat veterans with mood disorders

**Methods:**

A review of the literature on suicidal behavior in combat veterans with mood disorders including own publications.

**Results:**

Combat deployment may lead to multiple emotional, cognitive, psychosomatic symptoms, mood disorders, suicidal ideation and behavior. Pre-deployment, deployment and post-deployment adversities may increase risk of mood disorders and suicide in combat veterans. The act of killing in combat is a stressor which may raise suicide risk. Combat-related injuries are associated with significantly increased depression and suicide risk. Post-deployment difficulties of reintegrating into civilian life may lead to depression and suicidality. Studies suggest that suicidal behavior in combat veterans may have a neurobiological basis. Prevention of mood disorders and suicide among combat veterans should include pre-deployment screening to exclude individuals with psychiatric disorders; psychological support and prevention of harassment and/or abuse during deployment; psychosocial support after deployment; diagnosing and treating psychiatric and medical disorders including neurological disorders; frequent depression and suicide screening; education of mental and non-mental health clinicians, war veterans, their families and friends regarding signs/symptoms of mood disorders and suicidality; and restriction of access to lethal means.

**Conclusion:**

Combat veterans are a unique population. They are frequently exposed to psychological, physical, and biological factors which are unusual for civilians or non-combat military veterans. We need to study the specific psychobiology of combat veterans to understand how to develop effective depression and suicide prevention interventions for this population.

**Disclosure of Interest:**

None Declared

